# *In vivo* safety prediction of recombinant collagen using *in vitro* simulated degradation analysis, chronic toxicity and immunological evaluation

**DOI:** 10.1093/rb/rbaf128

**Published:** 2025-12-09

**Authors:** Can Huang, Jie Li, Xueyimu Aou, Hongwei He, Yun Chu, Jianping Gao, Hao Le, Xinhui Wang, Huilin Sun, Huimin Wang, Ziqing Zhu, Yujiang Fan, Guifeng Zhang, Xiaoju Fan, Tun Yuan

**Affiliations:** National Engineering Research Center for Biomaterials, Sichuan University, Chengdu 610064, China; College of Biomedical Engineering, Sichuan University, Chengdu 610064, China; Jiangsu Trautec Medical Technology Co., Ltd, Changzhou 213200, China; National Engineering Research Center for Biomaterials, Sichuan University, Chengdu 610064, China; College of Biomedical Engineering, Sichuan University, Chengdu 610064, China; National Engineering Research Center for Biomaterials, Sichuan University, Chengdu 610064, China; College of Biomedical Engineering, Sichuan University, Chengdu 610064, China; Jiangsu Trautec Medical Technology Co., Ltd, Changzhou 213200, China; Key Laboratory of Biopharmaceutical Preparation and Delivery, Institute of Process Engineering, Chinese Academy of Sciences, Beijing 100190, China; Jiangsu Trautec Medical Technology Co., Ltd, Changzhou 213200, China; Jiangsu Trautec Medical Technology Co., Ltd, Changzhou 213200, China; Jiangsu Trautec Medical Technology Co., Ltd, Changzhou 213200, China; Jiangsu Trautec Medical Technology Co., Ltd, Changzhou 213200, China; Jiangsu Trautec Medical Technology Co., Ltd, Changzhou 213200, China; National Engineering Research Center for Biomaterials, Sichuan University, Chengdu 610064, China; College of Biomedical Engineering, Sichuan University, Chengdu 610064, China; Key Laboratory of Biopharmaceutical Preparation and Delivery, Institute of Process Engineering, Chinese Academy of Sciences, Beijing 100190, China; College of Biomedical Engineering, Sichuan University, Chengdu 610064, China; Jiangsu Trautec Medical Technology Co., Ltd, Changzhou 213200, China; National Engineering Research Center for Biomaterials, Sichuan University, Chengdu 610064, China; College of Biomedical Engineering, Sichuan University, Chengdu 610064, China; Sichuan Testing Centre for Biomaterials and Medical Devices, Chengdu 610064, China

**Keywords:** protein-based biomaterials, simulated degradation product analysis, immunotoxicological evaluation, chronic toxicity studies, recombinant type III collagen

## Abstract

Protein-based biomaterials, particularly collagen-derived materials, have been widely applied in medical devices and regenerative medicine due to their excellent biocompatibility and tissue repair-promoting functions. However, the degradation of these materials *in vivo* may trigger immune responses and other physiological reactions, especially through interactions between degradation products and the immune system. To better evaluate their safety and efficacy, particularly regarding the potential immunotoxicological risks posed by degradation products, this study proposes a comprehensive evaluation framework combining degradation product simulation, immunotoxicological assessment and chronic toxicity testing, aiming to more fully identify potential risks during the degradation process. This study first simulated the degradation process *in vitro*, analyzing the compatibility of the resulting degradation products with human proteins to reveal potential molecular interaction risks. Based on this, a systematic immunotoxicological evaluation was conducted from multiple dimensions, including complement activation, humoral immunity, cellular immunity and inflammatory responses, to thoroughly assess the immunological safety of the material. Furthermore, chronic toxicity experiments confirmed the long-term biosafety and stability of the material. The results demonstrate that the established comprehensive evaluation framework provides new methodological support and reference for the *in vivo* long-term biological risk assessment. Using this framework, recombinant type III collagen was found to exhibit favorable biosafety and immunological compatibility at the molecular, immune and systemic toxicity levels.

## Graphical abstract

**Figure:**
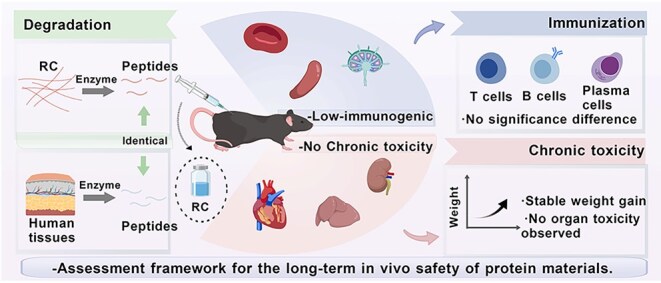

## Introduction

Protein-based biomaterials, particularly collagen-derived materials, are widely utilized in medical devices and regenerative medicine due to their exceptional biocompatibility and ability to promote tissue repair [1–4]. These materials closely resemble the body’s own protein structures, typically exhibiting low immune rejection *in vivo*—a key factor contributing to their favorable biocompatibility [5, 6]. However, after implantation, the degradation process of these materials is complex, and their degradation products may interact with the host, potentially triggering immune responses and other physiological reactions [7, 8]. Consequently, the safety and functional performance of these materials depend not only on their inherent properties but also on their dynamic changes within the body, particularly the potential for degradation products to induce immune responses [9–12]. Therefore, despite the broad clinical prospects of these materials, existing methods for evaluating their long-term safety and efficacy remain limited. To identify potential risks earlier and more rapidly, there is an urgent need to establish a rational, reproducible and predictive evaluation system for assessing the long-term safety of such materials [13].

Taking collagen-based materials as an example, they typically undergo progressive degradation processes *in vivo*, such as enzymatic degradation and hydrolysis, releasing products including peptide fragments and amino acids [14]. These degradation products engage in complex interactions with surrounding cells, the tissue microenvironment and the immune system [15, 16]. The degradation process is not only central to the material’s restorative functions—such as guiding cell migration, proliferation and differentiation [17]—but may also become a source of potential risks [18, 19]. Therefore, evaluating these materials requires addressing two key aspects: first, ensuring their efficacy, meaning they maintain their intended biological functions throughout degradation; second, guaranteeing their safety, particularly by focusing on immunotoxicological risks that may arise during degradation [20]. Immunotoxicological risks refer to how degradation products of research materials affect the immune system and may trigger adverse immune responses, including immune overactivation [21], chronic inflammatory reactions [22, 23] and loss of immune tolerance. These responses can sometimes lead to fibrosis [24], granuloma formation [25] and even serious health issues such as cancer [26–28]. Therefore, how to simultaneously consider the characteristics of degradation products and their immunological effects during the evaluation process has become a core issue that urgently requires resolution in research.

In current research practices, animal experiments remain a crucial method for evaluating material safety and efficacy, providing direct evidence of toxicity, immune responses and degradation-related effects following material implantation [27, 29, 30]. However, constrained by factors such as cost, observational methods and ethical considerations, the duration of animal experiments is inherently limited. This limitation makes it challenging to comprehensively track and assess all potential immune and toxic reactions throughout the collagen degradation process [31]. Although animal studies offer valuable preliminary insights, for highly cross-linked [32] or structurally complex materials, this timeframe may be insufficient to fully reflect their long-term degradation processes and the resulting immune responses [30, 33]. Therefore, recognizing this limitation, this study proposes an integrated approach combining chronic toxicity testing, simulated degradation product analysis and immunotoxicological evaluation. This aims to establish a more systematic assessment framework for comprehensively identifying *in vivo* long-term biological risks during material degradation at the preclinical stage.

*In vitro* experiments employed three enzymes—collagenase I, cathepsin B and cathepsin K—to simulate degradation processes. Each plays a distinct role in the human body: collagenase I efficiently degrades collagen, while cathepsin B and cathepsin K participate in breaking down more complex protein structures [34, 35]. Through the synergistic action of these enzymes, we can obtain peptide products resembling those generated during *in vivo* degradation. These products can then be analyzed for their matching patterns with human proteins, enabling assessment of their potential immunogenicity or carcinogenic risks.

Additionally, *in vivo* studies encompassed not only chronic toxicity experiments [36] to systematically observe the material’s overall toxicological effects during long-term implantation, but also comprehensive immunological evaluations [37, 38]. These focused on analyzing the material’s impact on immune cell function and inflammatory mediators [39, 40] across different degradation stages, thereby validating its immunological safety at the systemic level [41]. To comprehensively evaluate the potential impact of the material on the immune system during *in vivo* degradation, we employed a multi-dimensional, multi-time-point dynamic monitoring approach covering immune responses across different degradation stages. Specific evaluation aspects included:

1. Innate Immune Activation Level: Assess complement activation pathways and their impact on inflammatory responses by detecting changes in key complement system components (C3, C4, C5a);
2. Humoral Immune Response: Determine whether antigen-specific antibody responses are triggered and whether immune memory is formed by measuring levels of different immunoglobulin subclasses (IgM, IgG, IgA) [42];
3. Cell-mediated immunity and inflammatory status: Quantify Th1-type immune responses and inflammation intensity by analyzing the secretion profiles of key pro-inflammatory cytokines (e.g. IL-2, IL-6, IL-12p70, IFN-γ, TNF-α) [43];
4. Immune cell function and differentiation: Assess overall activation potential of immune cells through lymphocyte proliferation and transformation assays; Concurrently, conduct in-depth analysis of T cell (e.g. CD4^+^/CD8^+^ central memory T cells Tem, effector memory T cells Tcm) and B cell subsets (e.g. memory B cells memB, IgM-PC plasma cells and total CD19^+^ B cells) in peripheral blood, spleen and lymph nodes to reveal the long-term effects of material degradation products on adaptive immune cell homeostasis, memory cell generation and potential autoimmune predisposition [44, 45].

Through the integration of chronic toxicity studies, *in vitro* degradation simulations and *in vivo* immune assessments, we aim to elucidate the safety characteristics of materials throughout the entire degradation-metabolism-immune response process. This approach will provide methodological support and evaluation references for developing collagen-based medical devices with more complex structures or extended degradation cycles.

## Materials and methods

### Materials and reagents

Recombinant type III collagen (RC) was provided by Jiangsu Trautec Medical Technology Co., Ltd; China-approved recombinant collagen(A-RC) was purchased from Shanxi Jinbo Biopharmaceutical Ltd.

Collagenase I was purchased from MP Biomedicals, Inc. in the USA. Cathepsin K and calcium chloride were purchased from Sigma-Aldrich Corporation in the USA. Cathepsin B was purchased from Beijing Solarbio Science & Technology Co., Ltd. Acetonitrile and methanol were purchased from Thermo Fisher Scientific Inc. in the USA. Formic acid was purchased from Shanghai Aladdin Bio-Chemical Technology Co., Ltd. Trimethylolpropane was purchased from Shanghai Yien Chemical Technology Co., Ltd. Hydrochloric acid was purchased from Sinopharm Chemical Reagents Co., Ltd. 0.9% sodium chloride injection was purchased from Sichuan Meidakanjiale Pharmaceutical Co., Ltd.

Luminex assay kits, FITC anti-mouse CD3 antibody (17A2) and APC anti-mouse CD4 antibody (GK1.5) were purchased from Absin. ELISA kits, anti-mouse CD138 antibody (281-2) and blocker purchased from BD. PE/Cyanine7 anti-mouse CD8a purchased from Biolegend. PE rat PE/Dazzle™ 594 anti-mouse CD273 (B7-DC, PD-L2) were purchased from Biolegend. ANTI-HU/MO CD44 IM7 EF450, ANTI-M CD62L MEL-14 PCP-CYN5.5, anti-mouse CD19 EBIO1D3 BV650 and anti-mouse IgM II/41 SB600 were purchased from Thermo.

### Instrumentation

Liquid chromatograph (U3000) and high-resolution mass spectrometer (Obitrap Exploris 480) purchased from Thermo Fisher Scientific Inc. in the USA.

Luminex (Luminex X-200) purchased from Thermo Fisher. Centrifuge (5424R) was purchased from Eppendorf. Shaker (MTS 2/4) purchased from IKA GmbH, Germany. Electronic balance (Scout SE-SE601F) was purchased from Ohaus. Analytical balance (TP-114) was purchased from Denver. Fully automatic blood biochemistry analyzer (BS360S) was purchased from Mindray. Multi-functional enzyme-linked immunosorbent assay reader (SYNERGY LX) was purchased from BioTek. Refrigerated centrifuge (MICRO 17R) was purchased from Thermo. Flow cytometer (CytoFlex S) was purchased from Beckman.

### Ethics approval and consent to participate

The experimental animals included C57BL/6 JGpt mice (SPF grade) and H11-mCol3a1-hCOL3A1-WPRE-PolyA transgenic mice (SPF grade), all purchased from Chengdu YaoKang Biotechnology Co., Ltd, license number: SCXK(Sichuan)2020-0034. All procedures were conducted in accordance with relevant animal welfare guidelines.

The experimental animals used in chronic toxicity studies were SD rats (SPF grade) purchased from Sichuan Weitong Lihua Experimental Animal Technology Co., Ltd, license number: SCXK(Sichuan)2023-0040.

### *In vitro* degradation simulation

According to the method described by Gao *et al.* [46], tissue samples were first homogenized and freeze-dried after pretreatment with chloroform–methanol extraction and acetic acid soaking. Precisely 5 mg of lyophilized samples from human facial skin, foreskin, amniotic membrane and recombinant type III humanized collagen fibers were weighed using an analytical balance and transferred into 15-mL centrifuge tubes. Each sample was dissolved in 2 mL of 0.1 mol/L Tris–HCl buffer (pH 8.0, containing 10 mM CaCl_2_). Subsequently, 100 μg of collagenase I (dissolved in 0.1 mL of Tris–HCl buffer containing 10 mM CaCl_2_) was added, and the mixture was incubated at 37°C in a shaking water bath for 48 h.

After the initial digestion, 100 μg of proteinase K (dissolved in 0.1 mL Tris–HCl buffer containing 10 mM CaCl_2_) was added, and the reaction continued for another 48 h at 37°C. Then, 100 μg of proteinase B (dissolved in 0.1 mL Tris–HCl buffer containing 10 mM CaCl_2_) was introduced, and the mixture was incubated at 37°C for a further 48 h. The enzymatic reaction was terminated by adding 10% formic acid to a final concentration of 0.1%. After centrifugation, the supernatant was collected for liquid chromatography–mass spectrometry (LC-MS) analysis.

High-resolution LC-MS analysis was performed using an Orbitrap Exploris 480 mass spectrometer coupled with an Ultimate 3000 UHPLC system (Thermo Fisher Scientific, USA). A 5 μL aliquot of each digested sample was injected onto a Peptide CSH C18 column (1.0 × 150 mm, 1.7 μm; Waters) maintained at 60°C. Mobile phase A consisted of water containing 0.1% formic acid, and mobile phase B consisted of 60% acetonitrile with 0.1% formic acid. The gradient elution program was as follows: 0 min, 5% B; 80 min, 45% B; 90 min, 90% B; 100 min, 90% B; 110 min, 5% B; and 120 min, 5% B, with a flow rate of 0.1 mL/min.

Mass spectrometric conditions were as follows: spray voltage, 3.5 kV; capillary temperature, 320°C; sheath gas flow rate, 19.8 mL/min; auxiliary gas pressure, 5 psi. The instrument operated in positive ion mode. Scan event 1 was a full MS scan (m/z 300–2000) at a resolution of 60 000, RF Lens 45%, normalized AGC target 300% and maximum injection time 100 ms. Scan event 2 was data-dependent MS/MS acquisition with a resolution of 15 000, isolation window m/z 1.6, maximum injection time 200 ms, normalized AGC target 100%, normalized collision energy 30% and a duty cycle of 2 s.

The acquired data were processed and analyzed using BioPharma Finder and Proteome Discoverer software (Thermo Fisher Scientific).

### *In vivo* immunological tests

#### Information and groups of animals

The study consisted of three experimental cohorts, each comprising eight groups. Experiment I included wild-type mice; Experiment II included transgenic mice and Experiment III consisted of wild-type mice with an extended observation period. Within each cohort, eight groups were established: the experimental groups RC (RC-L, RC-M and RC-H, corresponding to low-, medium- and high-dose RC treatments, respectively); the China-approved recombinant collagen control groups A-RC (A-RC-L, A-RC-M, A-RC-H); and two control groups—a normal saline control group (NC) and a bovine serum albumin control group (BSA). The first day of administration was designated as Day 0 (D0). In Experiment I, each group contained ten C57BL/6JGpt wild-type mice. In Experiment II, each group contained four humanized transgenic mice. In Experiment III, each group contained six C57BL/6JGpt wild-type mice.

#### Administration

Without administering anesthesia, after the mice were fully sedated, two subcutaneous injections were performed on both sides of the spinal column on the back (the distance between the points was no less than 0.5 cm). Half of the total liquid volume was injected at each point, totaling 100 μL. The dosage for the NC group was 1.23 mL per animal, for the BSA group, it was 0.12 mL per animal, and for the RC group, the dosages were 0.08 mg per animal, 0.135 mg per animal and 4.92 mg per animal, respectively. For the A-RC group, the dosages were 0.02 mg per animal, 0.035 mg per animal and 2.46 mg per animal, respectively. The body weight of each mouse was recorded weekly.

Each animal was administered the drug via subcutaneous injection at the back of the spine on both sides at two points on D0, D30 and D60 according to the grouping scheme and at the set doses, totaling three injections. Blood collection time points for Experiment 1 and Experiment 2 were: D0, D15, D30, D45, D60, D75 and D90; For Experiment 3, the blood collection time points were: D0, D120, D150 and D180. The collected blood samples were subjected to subsequent testing.

#### Antibody and complement

The ELISA method was used to detect the levels of antibodies (IgA, IgG, IgM) and complement components (C3, C4, C5). The collected whole blood was separated for the serum/plasma by centrifuging at 4°C and 3000 g for 20 min, then the supernatant was collected. Fifty microliters of blocking solution was added to each well, then 50 μL of standard solution and sample were added, with a plate cover covering, and incubated at room temperature for 2 h. Then, the plate was washed three times with 400 μL of 1× dilution solution, and the last wash was performed by blotting on a blotting paper to dry. One hundred microliters of detection antibody was added to each well, with a plate seal covering, and was incubated at room temperature for 1 h. After incubation, the plate was washed three times with 400 μL of 1× dilution buffer, and the last wash was performed by blotting on a blotting paper to dry. Add 100 μL of TMB substrate solution to each well and incubate in the dark for 30 min. Then add 100 μL of stop solution to each well to terminate the reaction. Read at 450 nm, with 570 nm as the correction wavelength.

#### Cell phenotyping

After resuspending blood cell samples with lysing red blood cells, flow cytometry was used to detect the proportions of peripheral blood CD4 central memory T cells (Tcm/CD4), CD4 effector memory T cells (Tem/CD4), CD8 central memory T cells (Tcm/CD8), CD8 effector memory T cells (Tem/CD8), B cells (CD19+B/CD3−), memory B cells (memB/CD19) and plasma cells (IgM-PC).

At the end of the experiment, blood was collected from the orbital veins of each group of mice (anticoagulated blood) before euthanasia, and lymph nodes were collected from the inguinal region. After thorough grinding and hemolysis, the proportions of CD4 central memory T cells (Tcm/CD4), CD4 effector memory T cells (Tem/CD4), CD8 central memory T cells (Tcm/CD8), CD8 effector memory T cells (Tem/CD8), B cells (CD19+B/CD3−), memory B cells(memB/CD19) and plasma cells (IgM-PC) in the spleen and lymph nodes were detected by flow cytometry. The remaining animal carcasses (trunk and organs) were stored at −80°C for use in flow cytometry analysis.

#### Splenic lymphocyte transformation and proliferation

Euthanize the mice, aseptically remove the spleen and prepare a splenic lymphocyte suspension. The lymphocyte suspension, taken from each animal, was washed with PBS at least twice. The supernatant was discarded, and the cells were resuspended in 9 mL of PBS, adding 4 mL of CFSE (final concentration of 5 μmol/L) to each sample, mixing thoroughly. Incubate at 37°C in the dark for 10 min. Add 6 ml of pre-chilled RPMI 1640 medium containing 2% fetal bovine serum (FBS) to terminate the staining reaction, then gently mixed and centrifuged at 1500 rpm for 5 min. The supernatant was discarded, and then 4 mL of RPMI 1640 medium containing 10% FBS was added, with mixing thoroughly.

Add the cell suspension to a 24-well plate according to the above groups, 1 mL per well. Add the corresponding ConA to the stimulation group at a final concentration of 5 μg/mL. Incubate the test plate at 37°C in 5% CO_2_ for 3 days. Collect cells from each well, and stain with PE-labeled anti-mouse CD3 monoclonal antibody, incubate at 4°C in the dark for 15 min and analyze by flow cytometry.

#### Cytokine

The collected whole blood was separated for the serum/plasma by centrifuged at 4°C and 3000 g for 20 minutes, then the supernatant was collected. The levels of cytokines (IL-2, IL-6, IL-12 P70, TNF-α, IFN-γ) were detected according to the Luminex kit instructions.

#### Statistical analysis

The experimental results are expressed as mean±SEM. For comparisons between multiple groups, one-way analysis of variance (ANOVA) was used. For comparisons between multiple groups at different time points, two-way ANOVA was used. All data were analyzed using GraphPad Prism. *P* < 0.05 was considered to indicate a statistically significant difference.

### Chronic toxicity studies

#### Information and groups of animals

Referring to GB/T 16886.11-2021 ‘Biological Evaluation of Medical Devices—Part 11: Systemic Toxicity Tests’, 80 SD rats (equal numbers of males and females) were randomly divided into two groups: the RC group (20 rats of each sex) and the NC group (20 rats of each sex). RC group: Intradermal injection of the reconstituted sample (8 mg/mL). Each animal was injected at 10 injection sites on either side of the midline of the back, with 0.1 mL injected at each site, resulting in a single total dose of 1 mL per animal. A total of three injections were administered (the first injection was on Day 1, with subsequent injections on Days 31 and 61), resulting in a cumulative dose of 3 mL per animal. NC control group: Equal volumes of physiological saline were injected using the same method. The observation period was 180 days, starting from the third injection. After the observation period, all animals were weighed and fasted for 12–18 h.

#### Clinical observations

Observe and record the general condition of each group of animals weekly, including weight, behavior, coat, mucous membranes, respiration, nervous system, etc, noting the time, severity and duration of any changes. For details, refer to Appendix C of GB/T 16886.11-2021 ‘Biological evaluation of medical devices—Part 11: Tests for systemic toxicity’ [36].

#### General pathology

At the end of the experiment, after completing blood sampling from the abdominal aorta, a gross postmortem examination was performed on all animals, including examination of the body surface, body orifices, head, thoracic cavity, abdominal cavity and internal organs. The wet weights of major organs, such as the heart, liver, spleen, lungs, kidneys, adrenal glands, gonads, brain and thymus, were promptly measured and recorded, along with any pathological changes observed in each organ.

#### Histopathology

Take the hearts, livers, spleens, lungs, kidneys, adrenal glands, thymus glands, brains (cerebrum, cerebellum, brainstem), stomachs, ovaries, uteruses (uterine cervix and fallopian tubes), testes, epididymis, sternum, muscles, skin, intestines (duodenum, jejunum, ileum, colon), eyes, trachea, esophagus, pancreas, bladder, mammary glands, sciatic nerve, lymph nodes (mesenteric), thyroid gland (parathyroid glands), vagina, prostate, salivary glands, etc, were fixed in 10% neutral formalin solution and examined under a microscope after Hematoxylin and Eosin (H&E) staining.

## Results and discussion

This study aims to establish a comprehensive *in vitro*-*in vivo* correlation evaluation system to assess the immunotoxicological risks of collagen-based biomaterials during long-term degradation. Through chronic systemic toxicity testing of RC over 34 weeks, *in vitro* analysis of simulated degradation products, and multi-time point, multi-dimensional immunotoxicological evaluations, we obtained systematic data on the long-term safety of this material. The experimental results overall indicate that the material exhibits good biocompatibility and extremely low immunogenicity risk.

### *In vitro* degradation simulation

**Figure 1:**
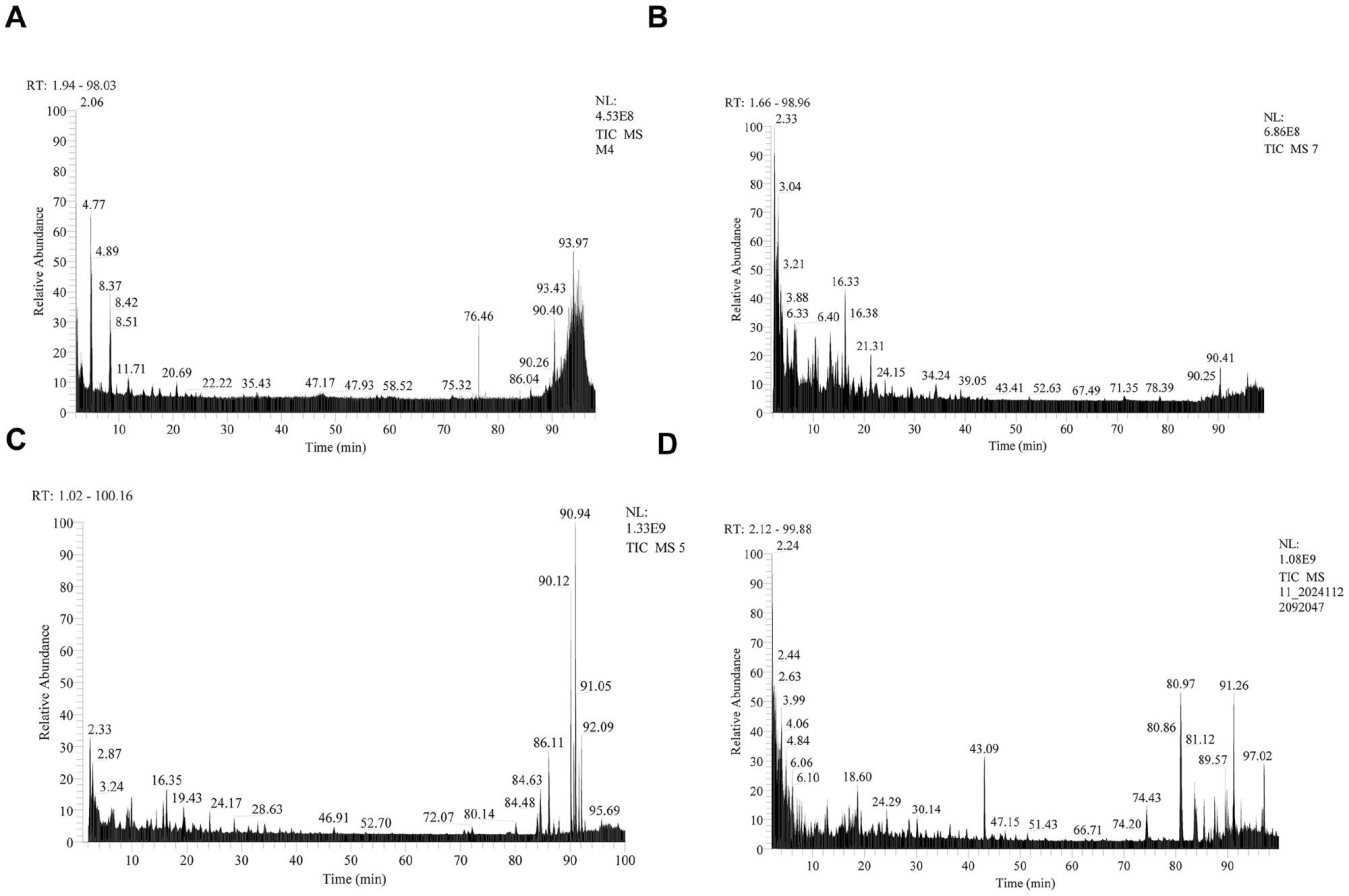
Total ion chromatograms of enzymatic hydrolysis products from different samples. (**A**) RC, (**B**) human facial skin, (**C**) human foreskin and (**D**) human amniotic membrane.

To determine whether the peptides produced by RC hydrolysis are detected in the hydrolysis products of human tissues, LC-MS was used to analyze the hydrolysis products, and mass spectra were obtained for hydrolysis products from RC, human facial skin, foreskin and amniotic membrane (Figure 1). The sequence information of RC was imported into the BioFinder software to construct a database, and the mass spectrometry data of the RC enzymatic hydrolysis products were searched based on this database to obtain the peptide sequences after enzymatic hydrolysis. Then, the human proteome database was downloaded from the Uniprot website (https://www.uniprot.org/uniprotkb? query=human&facets=reviewed%3Atrue), imported into the Proteome Discoverer software and used to analyze the enzymatic hydrolysis products of human facial skin, foreskin and amniotic membrane tissues, yielding the peptide sequences from the enzymatic hydrolysis products of these three tissues.

The peptide sequences of RC were compared with those obtained from the enzymatic hydrolysis of human facial skin, foreskin and amniotic membrane tissues. The results showed that the sequence coverage of RC relative to these tissues was 79.70% for human facial skin, 81.21% for foreskin and 77.97% for amniotic membrane. Based on the match with human facial skin, further matching analysis was conducted with the enzymatic hydrolysis products of foreskin and amniotic membrane, achieving a match rate of 89.63%. The primary reason for the differences in peptide match rates may be that the protein composition, relative abundance and content vary among different human tissues. These variations further lead to differences in the peptide composition produced after enzymatic hydrolysis, thereby influencing the final matching results.

The RC was compared with peptide segments that did not match in any of the three tissues and with the entire human protein database (Table 1). Some individual K (lysine) residues failed to match, possibly due to the random nature of proteasome cleavage, resulting in residual individual amino acids that could not be successfully matched after cleavage [47]. Additionally, the proteins corresponding to the undetected peptide segments in Table 1 have relatively low abundance in the human body. Combined with the limited sensitivity of mass spectrometry detection and the inhibitory effect of high-abundance peptides, this makes it difficult to effectively detect, resulting in their low-abundance peptides.

**Table 1:** Matching results of peptide segments that do not match human tissue enzymatic products with the entire human protein database.

Sequence	Location	Matching proteins and locations
K	25, 105, 184, 253, 333 and 412	K is present in various proteins in human tissue.
KGESGK	70–75, 298–303	Human type III collagen (977–982)
AGPAGA	163–168, 391–396	Human type III collagen (1070–1075), human type I collagen (α2 chain, 724–729), etc.
TGEK	190–193, 418–421	Human type IV collagen (α5 chain, 937–940; α6 chain, 284–287), human type IV collagen (α6 chain, 284–287), human type V collagen (α2 chain, 82–815; α3 chain, 750–753), human type XII collagen (948–951), human type XVI collagen (395–398, 817–820), human type XIX collagen (339–342), human type XXV collagen (299–302, 493–496), human type XXVI collagen (225–228), etc.
KGHK	199–202, 427–430	Human type IV collagen (α4 chain, 565–568), human type XI collagen (α2 chain, 732–735), human type XIX collagen (306–309) and human type XXVII collagen (708–711), among others.
HHHHHH	458–463	Human FOX protein (47–52), DYRK protein (607–612)

In summary, the simulated degradation results demonstrated that the peptide sequences resulting from RC degradation exhibit high consistency with the human tissue proteome, enabling complete matching. This finding indicates that RC possesses a relatively low risk of immunogenicity [48, 49].

### *In vivo* immunological experiments

#### Antibody

The IgA, IgG and IgM levels of RC-L, RC-M and RC-H did not show statistically significant differences from the NC group at any time point within 90 days after the first injection (*P* > 0.05).

**Figure 2:**
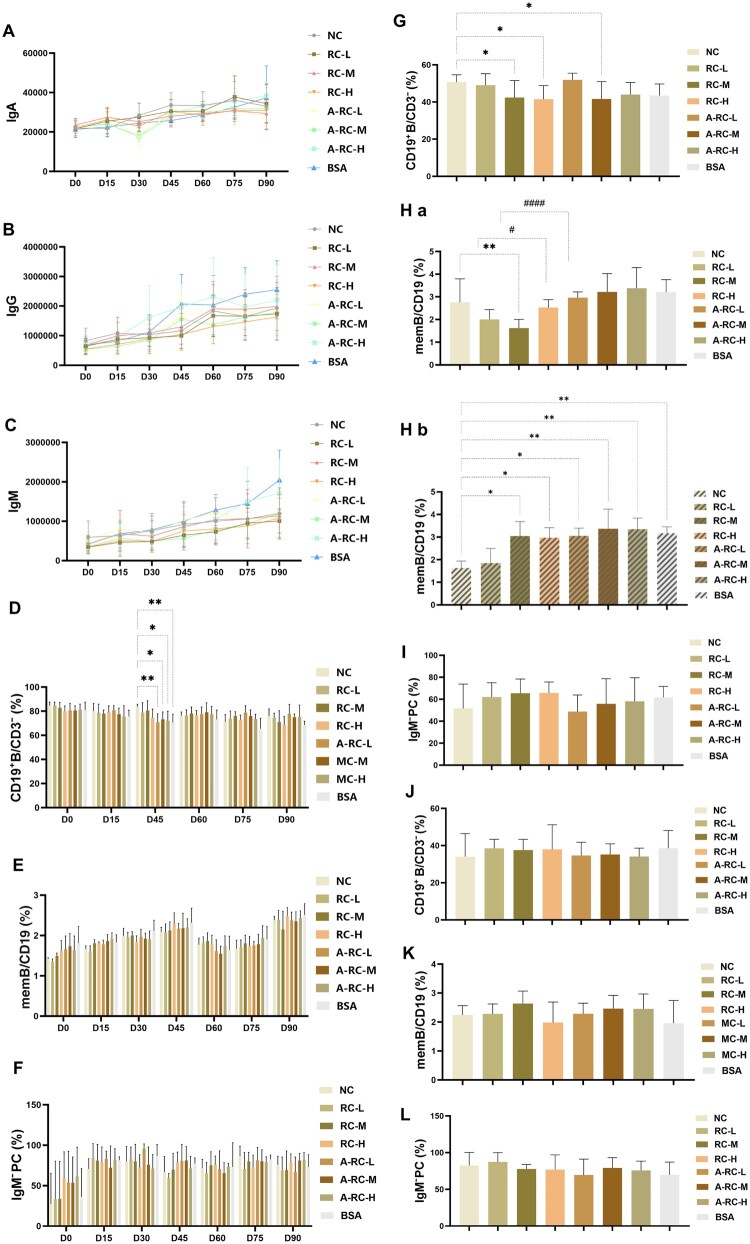
Humoral immunity. Antibody levels at different time points: (**A**) IgA, (**B**) IgG and (**C**) IgM; peripheral blood cells at different time points: (**D**) CD19+/CD3−, (**E**) memB/CD19 and (**F**) IgM-PC ratio; spleen at 90 days post-first injection: (**G**) CD19+B/CD3−, (**H**) memB/CD19 and (**I**) IgM-PC ratio; (Ha) represents wild-type mice group, and (Hb) represents transgenic mice group; 90 days post-first injection, lymph nodes: (**J**) CD19+B/CD3−, (**K**) memB/CD19 and (**L**) IgM-PC ratio. Compared with the blank control group, *P < 0.05. Compared with the corresponding dose of the China-approved recombinant collagen, ^#^*P* < 0.05.

The IgA levels in the RC dose groups at 0–90 days showed a similar trend to those in the NC and A-RC groups (Figure 2A). The IgG and IgM levels in the RC dose groups at 0–90 days showed a slight increase, but the trend was consistent with that in the NC and A-RC groups (Figure 2B and C).

This suggests that RC does not induce the production of new antigen-specific antibodies in the humoral immune response, nor does it form persistent immune memory, which is consistent with the results of simulated degradation products showing ‘high matching of degradation peptides with human tissue proteins’, further supporting its relatively low immunogenicity risk.

#### B cell

The peripheral blood B-cell-related markers (CD19^+^B/CD3^−^, memB/CD19, IgM-PC) in the RC groups at all doses showed no statistically significant differences compared to the NC group (Figure 2D–F, *P* > 0.05).

In the spleen, the proportion of B cells (CD19^+^B/CD3^−^) in the RC-L group was comparable to that in the NC group (*P* > 0.05), while the RC-M and RC-H groups exhibited significantly lower levels than the NC group (*P* < 0.05), though no significant differences were observed between the corresponding doses of A-RC (*P* > 0.05) (Figure 2G).

For splenic memory B cells (memB/CD19), no differences were observed between the RC-L and NC groups (*P* > 0.05), but the RC-L group was lower than the corresponding A-RC-L group (*P* < 0.05). The RC-M group was significantly lower than both the NC group and the A-RC-M group (*P* < 0.05) (Figure 2H). In transgenic mice, the proportion of splenic memory B cells in the RC and A-RC groups at all doses was higher than that in the NC group (*P* < 0.05), but no significant differences were found between RC and A-RC groups (*P* > 0.05) (Figure 2Hb). The proportion of plasma cells (IgM-PC) in the RC groups at all doses showed no significant differences compared to the NC group (Figure 2I, *P* > 0.05). In the lymph nodes, there were no significant differences in the proportion of B cells, memory B cells or plasma cells between the RC groups and the NC group (Figure 2J–L, *P* > 0.05).

In summary, RC did not cause significant changes in the peripheral and lymph node B cell profile, nor was there a significant increase in the proportion of plasma cells, indicating that the test samples did not significantly activate B cell-related immune responses. The partial differences observed in the spleen may be mainly due to species-specific immune recognition background rather than the immunogenicity of RC itself. Combined with the lack of sustained elevation in serum antibodies, this suggests that RC did not exhibit significant humoral immune activation or long-term memory formation trends. This result is consistent with the negative chronic toxicity findings and molecular evidence of high similarity between degraded peptide fragments and human proteins, supporting the conclusion of low immunogenicity risk.

#### T cell

**Figure 3:**
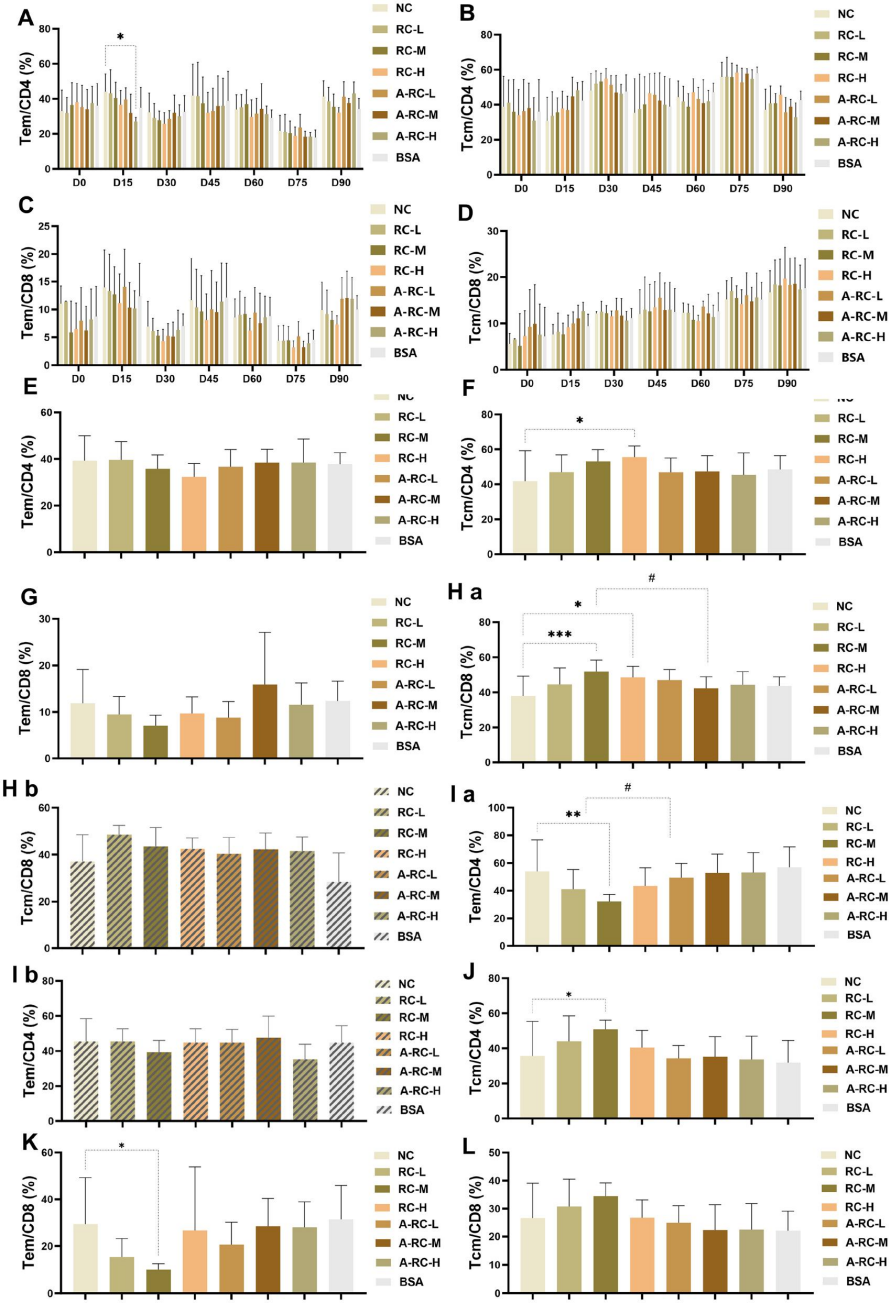
T lymphocyte immune typing. Proportions of peripheral blood (**A**) Tem/CD4, (**B**) Tcm/CD4, (**C**) Tem/CD8 and (**D**) Tcm/CD8 at different time points; spleen at 90 days post-first injection (**E**) Tem/CD4, (**F**) Tcm/CD4, (**G**) Tem/CD8 and (**H**) Tcm/CD8 ratios; In panel (H), (Ha) represents the wild-type mice group, and (Hb) represents the transgenic mice group; 90 days after the first injection, lymph nodes (**I**) Tem/CD4; (**J**) Tcm/CD4; (**K**) Tem/CD8 and (**L**) Tcm/CD8 ratio; In panel (**I**), (Ia) is the wild-type mice group, and (Ib) is the transgenic mice group. Compared with the blank control group, *P < 0.05. Compared with the corresponding dose of the China-approved recombinant collagen, ^#^*P* < 0.05.

There were no statistically significant differences in the proportion of T cell-related cells in peripheral blood between the RC dose groups and the NC group (Figure 3A–D, *P* > 0.05). In wild-type mice group, repeated exposure to RC did not result in peripheral blood immune memory that differed from that of the blank control.

The proportions of splenic Tcm/CD4^+^ and Tcm/CD8^+^ cells in the RC-H group were significantly higher than those in the NC group, but no statistically significant differences were observed compared with the corresponding A-RC-H group (Figure 3F and Ha, *P* > 0.05). The proportion of splenic Tcm/CD8^+^ cells in the RC-M group was significantly higher than that in both the NC group and the A-RC-H group (Figure 3Ha, *P* < 0.05). In transgenic mice, there was no significant difference in the proportion of splenic Tcm/CD8^+^ cells between the RC-M and NC groups (Figure 3Hb, *P* > 0.05).

The proportion of lymph node Tem/CD4^+^ cells in the RC-M group was significantly lower than that in the NC group and the A-RC-M group (Figure 3Ia, *P* < 0.05).

In transgenic mice, no significant difference in this cell population was observed between the RC-M and NC groups (Figure 3Ib, *P* > 0.05). For the RC-L and RC-H groups, the lymph node T-cell-related parameters (Tem/CD4^+^, Tem/CD8^+^ and Tcm/CD8^+^) showed no statistically significant differences compared with the NC group (Figure 3J–L, *P* > 0.05). Taken together, RC showed differences compared with the NC or A-RC groups in wild-type mice, whereas such differences disappeared in the chimeric mouse model, suggesting that these variations were likely attributable to species-specific background effects rather than to any intrinsic immunological activity of the material itself.

#### Splenic lymphocyte transformation and proliferation

**Figure 4:**
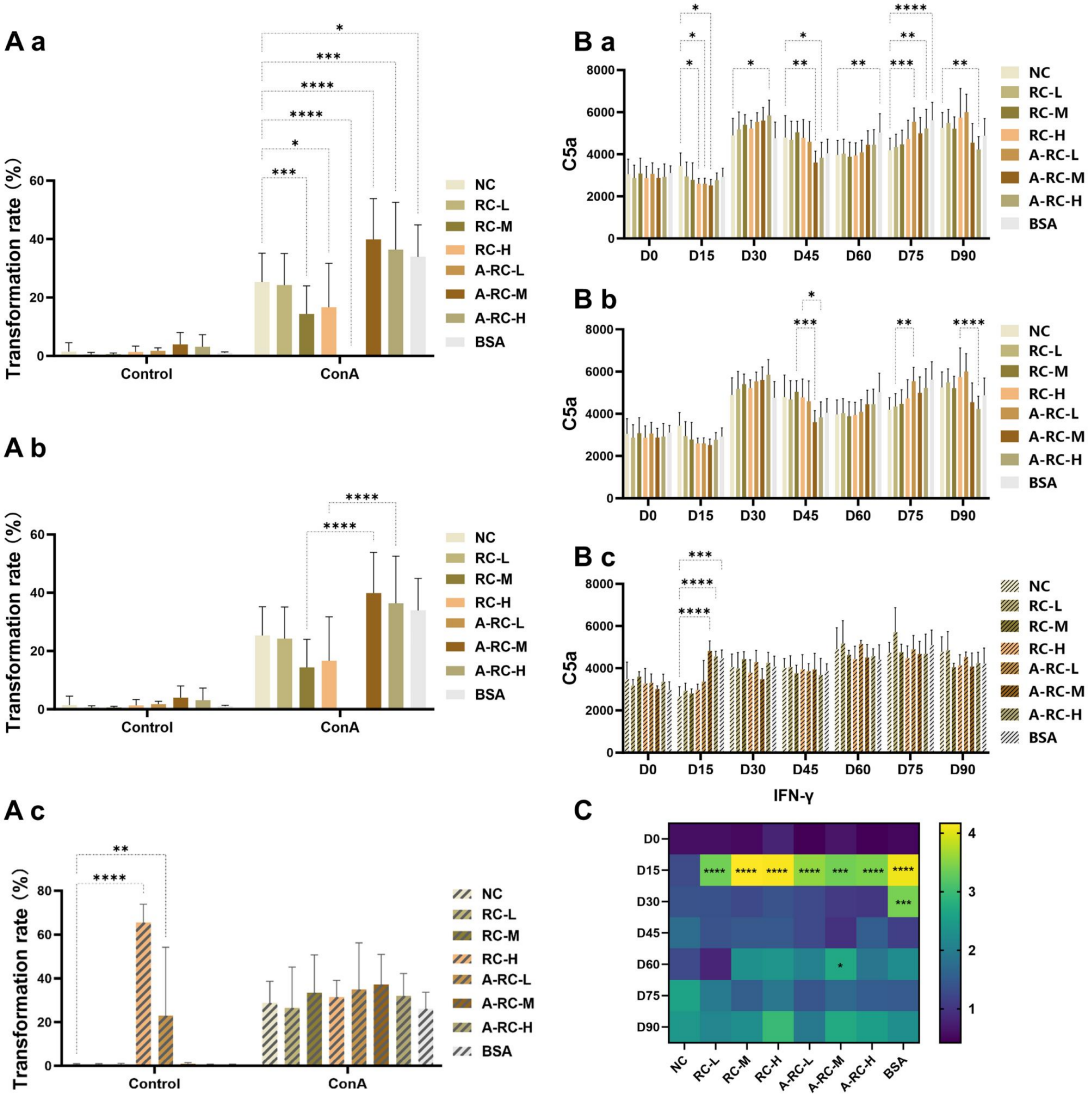
(**A**) Lymphocyte transformation and proliferation in mouse spleens 90 days after the first injection, where (Aa) represents the comparison between each experimental group and the blank control in the wild-type mouse experiment; (Ab) represents the comparison between each experimental group and the corresponding dose of the China-approved recombinant collagen in the wild-type mouse experiment; (Ac) represents the comparison between each experimental group and the blank control in the transgenic mouse experiment. Complement (**B**) C5a at different time points, where (Ba) represents wild-type mouse experiments, with each experimental group compared to the blank control; (Bb) represents wild-type mouse experiments, with each experimental group compared to the corresponding dose of the China-approved recombinant collagen; (Bc) represents transgenic mouse experiments, with each experimental group compared to the blank control. *P < 0.05. (**C**) IFN-γ concentrations at different time points. Compared with the blank control group, *P < 0.05; compared with the corresponding dose of the China-approved recombinant collagen, *P* > 0.05.

At 90 days post-injection, splenic lymphocyte proliferation in the RC-L group showed no significant difference compared with the NC group (*P* > 0.05). In contrast, the RC-M and RC-H groups exhibited significantly lower proliferation levels than the NC group (Figure 4Aa, *P* < 0.05), and also lower than those in the corresponding A-RC-M and A-RC-H groups (Figure 4Ab, *P* < 0.05). Meanwhile, splenic lymphocyte proliferation in the A-RC-M and A-RC-L groups was significantly higher than that in the NC group.

In transgenic mice, no statistically significant differences in splenic lymphocyte proliferation were observed between any RC dose group and the NC group (Figure 4Ac, *P* > 0.05). Taken together, the differences observed between the RC and NC or A-RC groups in wild-type mice disappeared in the transgenic model, suggesting that these variations likely originated from interspecies immune recognition differences rather than material-specific effects. Overall, the results indicate that RC did not elicit a specific T-cell immune response or induce sustained immune activation.

#### Complement

At all time points, the levels of C3 and C4 in the RC groups at different doses showed no significant differences compared with those in the NC group (Figure S1A and B, *P* > 0.05). Similarly, the C5a levels in the RC-L and RC-M groups did not differ significantly from those in the NC group at any time point (*P* > 0.05). However, on Day 15, the C5a level in the RC-H group was significantly lower than that in the NC group (Figure 4Ba, *P* < 0.05).

When compared with the A-RC groups (Figure 4Bb), no significant differences were observed between the RC groups and their corresponding A-RC groups on Days 0, 15 and 30 (*P* > 0.05). On Day 45, the C5a levels in the RC-M and RC-H groups were significantly higher than those in the corresponding A-RC groups (*P* < 0.05). On Day 75, the C5a level in the RC-L group was significantly lower than that in the A-RC-L group (*P* < 0.05), whereas on Day 90, it was significantly higher (*P* < 0.05).

In transgenic mice, no significant differences in C5a levels were found between any RC dose group and the NC group (Figure 4Bc, *P* > 0.05). Overall, although minor fluctuations in C5a levels were observed at individual time points, no persistent differences were detected, and these variations disappeared in the transgenic mouse model.

#### Cytokines

There were no statistically significant differences in the levels of IL-2, IL-6, IL-12p(70) and TNF-α between the RC dose groups and the NC group at all time points (Figure S1C–F, *P* > 0.05).

The levels of IFN-γ in the RC dose groups were significantly higher than those in the NC group at D15 (*P* < 0.05), but there were no statistically significant differences compared with the corresponding dose groups of the A-RC group (Figure 4C, *P* > 0.05).

Therefore, cytokine detection also indicated that RC did not exhibit persistent abnormal inflammatory responses.

Overall, both RC and A-RC exhibited comparable immunological profiles across multiple evaluation dimensions, including antibody levels, B cell and T cell subsets, splenic lymphocyte proliferation, complement activation and cytokine secretion. No abnormal immune activation or suppression was observed in either material. The consistency of these results indicates that both RC and A-RC possess stable immunological properties and acceptable biosafety, suggesting a low risk of immunogenicity under the tested conditions. Minor variations observed in individual indicators may be attributed to subtle differences in sequence design, dosage effects or species-related biological variability rather than intrinsic material immunogenicity.

This consistent immunological performance provides a solid foundation for evaluating the long-term biosafety of RC and A-RC in the subsequent chronic toxicity studies.

### Chronic toxicity studies

**Figure 5:**
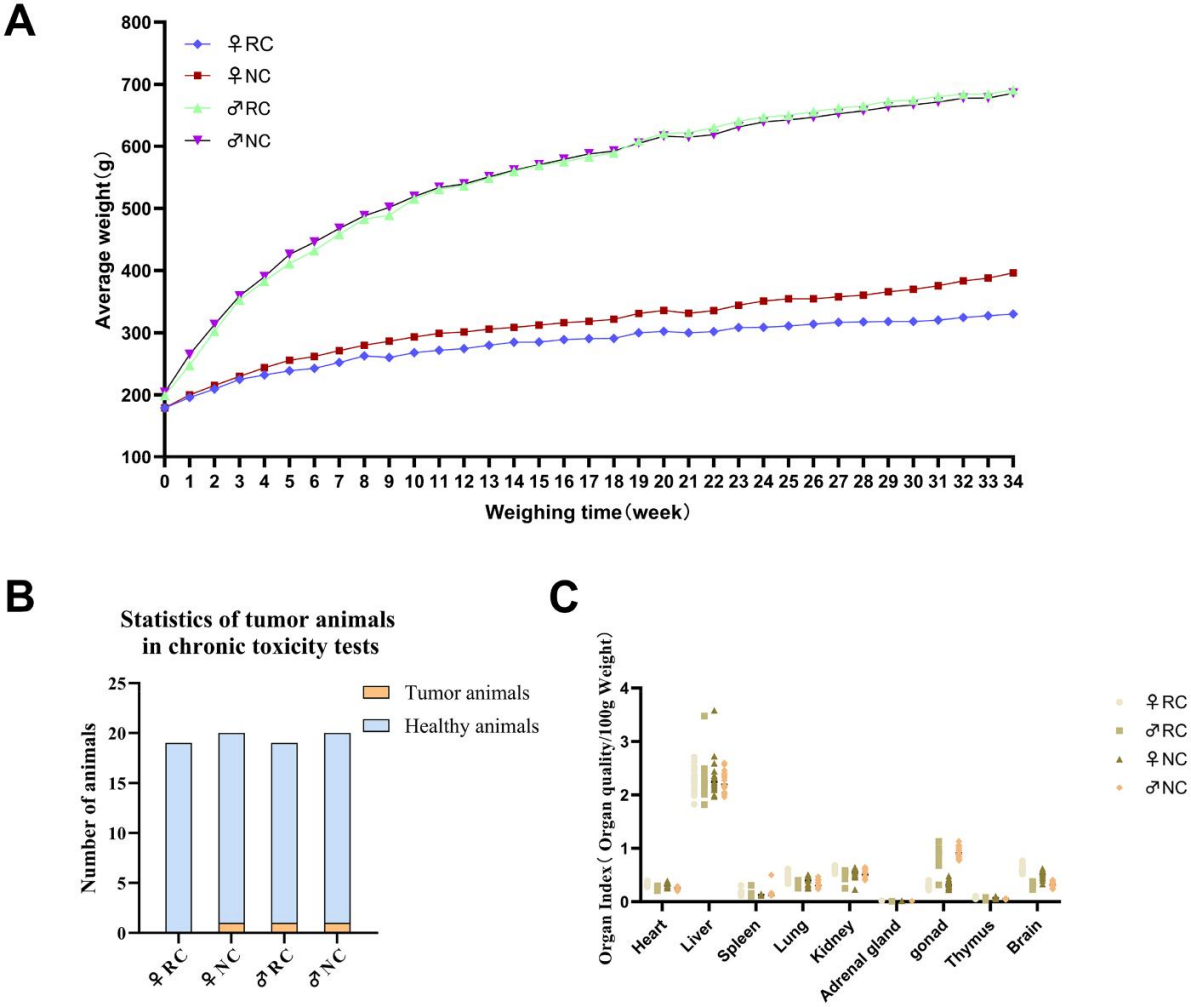
(**A**) Average weight change in experimental group animals and control group animals; (**B**) Statistics on tumor animals in the slow-poisoning test; (**C**) Summary of organ indices in experimental group animals and control group animals.

Repeated exposure systemic toxicity studies provide information on the health hazards associated with sustained exposure via the expected clinical route of exposure, as well as information on the toxicity patterns of the substance via the expected clinical route of exposure. During the 34-week feeding trial period, clinical observations showed that the behavior of animals in all trial groups was normal, and no toxic symptoms caused by the sample were observed. As shown in Figure 5A, the body weight of rats in all trial groups showed a continuous growth trend, and there was no significant difference in body weight between the trial group animals and the control group animals during the same period. During the same period, the weight gain of male rats was higher than that of female rats, which is consistent with the normal growth pattern of rats [50].

Macroscopic pathological findings and histopathological analysis revealed one tumor case in the male experimental group and one tumor-related case each in the female and male control groups (Figure 5B). Considering the high incidence of spontaneous tumors in middle-aged and elderly SD rats, as reported by Chao [51] in his data collection and analysis of SD rats: the tumor incidence rate in SD rats aged 52–104 weeks was 63.9%. A study by Marie Bockenstedt *et al.* [52] showed that in a 104-week study (where rats were euthanized if they were near death), the incidence of lymph-related diseases among spontaneous non-tumor lesions was 7.2% in males and 7.9% in females; the incidence of thymus-related diseases was 4.7% in males and 3.8% in females; and the incidence of mammary gland diseases was 11.8% in males and 72.3% in females. Based on clinical observations, clinical pathology, gross necropsy, and histopathological examination results, we conclude that these tumor cases were spontaneous tumors and not caused by abnormalities in the experimental samples.

Except for the experimental animals with spontaneous tumors, the major organs of the rats in each experimental group were of normal size, had good texture, intact surfaces, uniform color and no visible lesions. Organ indices are important indicators for evaluating the degree of organ development, as their size is related to rat growth metabolism and immune function, and they are also important indicators for evaluating drug toxicity [53]. The organ index results for each experimental group (Figure 5C) showed that, compared with the control group, there were no significant differences in organ coefficients among the experimental groups (*P* > 0.05), indicating that the experimental samples had no toxic effects on animal organs.

In addition, histological examinations of immune-related organs, including the spleen and thymus, were performed. The results showed that the tissue structures of all groups were intact, with orderly cellular arrangement and no signs of follicular disruption, necrosis or marked inflammatory infiltration. The morphology and histological features of immune organs in the experimental group remained within the normal range compared with the control group. These findings were consistent with the immunological analysis results, further supporting that RC did not induce systemic immunotoxicity or structural damage to immune organs under long-term exposure. The corresponding histological images are not included here because they are part of a separate ongoing study.

Overall, the immunological results are consistent with the chronic toxicity study and simulated degradation product analysis: the simulated degradation products indicate that the peptides resulting from RC degradation are highly compatible with human tissue proteins, thereby reducing the potential risk of immunogenicity; immunological evaluations confirmed across multiple levels, including humoral immunity, cellular immunity, complement and inflammatory factors, that RC does not induce new immunogenicity or long-term immune activation. The observed individual differences are primarily attributed to species background rather than material-specific effects; chronic toxicity experiments showed that long-term exposure to RC did not result in systemic toxicity or organ damage. Therefore, this study comprehensively validated RC’s good biosafety and immunological compatibility through simulated degradation product analysis, systematic immunological evaluation and chronic toxicity testing.

At the same time, we traced the sales situation and adverse event feedback of RC. As of now, approximately 320 000 bottles of RC have been sold, and they have been used in humans for 2 years without any adverse events related to the product caused by long-term *in vivo* interactions. This is consistent with the research results, indicating that the raw materials used in RC have good biocompatibility and immunological compatibility.

Building on this foundation, it is essential to revisit the original research objectives and reflect on the core safety challenges faced by complex protein materials in clinical applications, as well as the limitations of existing evaluation methods. The greatest challenge in the application of complex protein materials lies in their potential biological risks, including abnormal immune responses, interference with reproductive development and long-term carcinogenic threats. While existing carcinogenicity evaluation models are widely applied in the pharmaceutical field, they exhibit significant limitations when applied to medical devices: comparing tumor incidence rates in wild-type mice makes it difficult to distinguish whether the tumors are caused by the material itself or by immune disorders triggered by interspecies immune recognition differences; while constructing tumor-promoting or tumor-suppressing mouse models through genetic engineering can enhance sensitivity, it still cannot avoid interference from abnormal immune recognition, and tumor development often involves multi-gene networks, making it challenging to confirm causal relationships. In contrast, the comprehensive evaluation framework proposed in this study—combining simulated degradation product analysis, *in vivo* immunological evaluation and chronic toxicity testing—not only provides a new methodological approach for assessing the immunological safety of complex protein materials but also offers a reference framework for evaluating the potential carcinogenic risks of such materials in the future. Simulated degradation product analysis demonstrated that the degradation peptides highly match endogenous human proteins, thereby reducing immunogenicity risks; immunological experiments validated that the material does not induce new immune abnormalities across multiple dimensions, including humoral immunity, cellular immunity, complement and inflammatory factors; chronic toxicity results indicated that long-term exposure to the material did not induce systemic toxicity. Through multi-level evidence from the molecular to the systemic level, this study not only addresses the core scientific issues in preclinical safety evaluation of complex protein materials but also provides methodological support for establishing a more scientific and scalable safety assessment system for medical devices in the future, particularly in terms of *in vivo* long-term biological risk assessment, offering new pathways.

At the same time, we recognize that the broader application of this evaluation framework requires alignment and comparison with existing international regulatory systems to further emphasize its scientific innovation and practical relevance. Therefore, we analyzed the applicability and limitations of current ISO and OECD standards in the context of immunotoxicity assessment.

We fully acknowledge the importance of existing international standards in guiding immunotoxicological evaluations. The OECD test guidelines provide a well-established framework for assessing the immunotoxicity of chemical substances, while ISO 10993-20 offers a directional framework for the immunotoxicity assessment of medical devices. These standards have played a vital role in promoting regulatory harmonization and ensuring the safety of biomedical products.

However, when applied to specific classes of biomaterials—such as recombinant collagen-based medical devices—these frameworks present certain practical limitations. The OECD guidelines primarily focus on xenobiotic chemicals and their capacity to elicit systemic immune responses. In contrast, recombinant collagen undergoes metabolic degradation into endogenous peptides that naturally exist in the human body. Therefore, testing paradigms and long-term immune monitoring approaches designed for chemical substances are not fully suitable for evaluating protein-based biomaterials.

Similarly, ISO 10993-20 provides general principles for immunotoxicity assessment of medical devices but lacks detailed methodologies for evaluating long-term immune responses, immune tolerance and safety in protein-derived materials.

To address these limitations, this study proposes a comprehensive immunological safety evaluation framework specifically tailored for recombinant collagen-based medical devices. By integrating simulated degradation product analysis with *in vivo* immunotoxicity assessments and long-term subchronic toxicity studies, this approach provides a more holistic understanding of the biological behavior and immunological impact of RC degradation products. These findings offer methodological insights and a new perspective for the clinical application and regulatory evaluation of protein-based biomaterials.

It should be noted that no preclinical model can fully recapitulate the complexity of the human immune system, particularly under long-term exposure conditions. The results of this study demonstrate that RC exhibits excellent immunological compatibility and biosafety; however, these conclusions should be further verified through long-term clinical follow-up and real-world evidence.

## Conclusion

This study first identified through simulated degradation product analysis that the peptide sequences resulting from the degradation of RC materials exhibit high similarity to endogenous human proteins, suggesting a low immunogenicity risk at the molecular level. Further immunological evaluation revealed that RC materials did not induce new immune responses or long-term immune activation in multiple aspects, including humoral immunity, cellular immunity, complement and inflammatory factors. The observed individual differences primarily stemmed from species-specific background effects. Chronic toxicity tests confirmed that RC materials did not cause systemic toxicity or organ damage under long-term exposure conditions. In summary, RC materials exhibit a low risk of immunogenicity and toxicity at the molecular, immunological and overall toxicity levels. Although preclinical models cannot fully replicate the complexity of the human immune system, the integrated evaluation framework established in this study—combining simulated degradation analysis, immunological assessment and subchronic toxicity testing—offers a physiologically relevant and broadly applicable approach for assessing the long-term safety of complex protein-based biomaterials, providing valuable guidance for their clinical and regulatory development.

## Supplementary Material

rbaf128_Supplementary_Data

## Data Availability

The data are available from the corresponding authors upon reasonable request.
