# Supplementary material for: In vivo safety prediction of recombinant collagen using in vitro simulated degradation analysis, chronic toxicity and immunological evaluation
Source: Regen Biomater. 2025 Dec 9;13:rbaf128. doi: 10.1093/rb/rbaf128 (PMC12900536; doi:10.1093/rb/rbaf128)
Supplement: rbaf128_Supplementary_Data [file rbaf128_supplementary_data.docx]

**Supporting：**


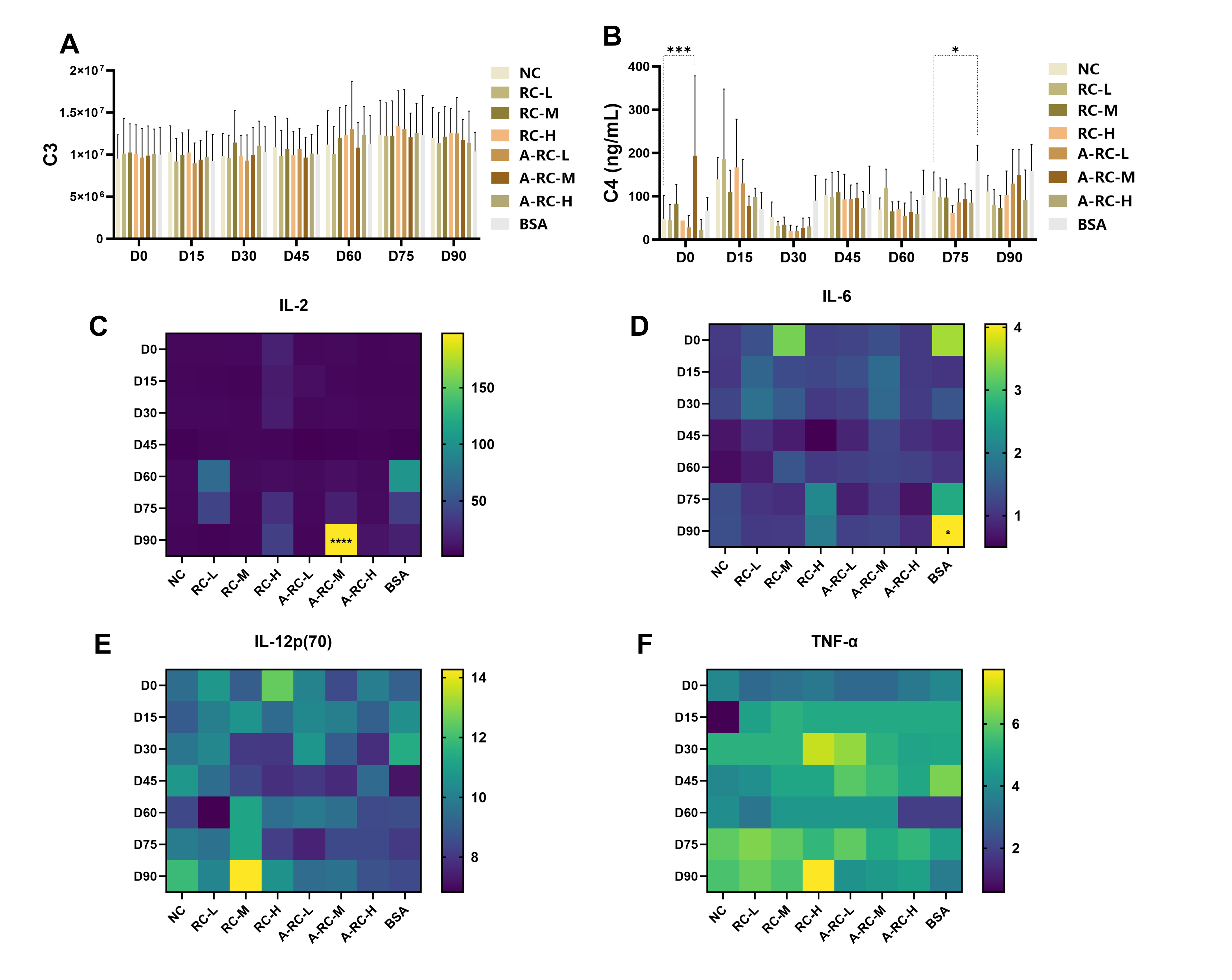


Figure S1. Complement levels at different time points: A. C3 and B. C4. *p<0.05. Concentrations of cytokines at different time points: C. IL-2, D. IL-6, E. IL-12p(70), and F. TNF-α. Compared with the blank control group, *p < 0.05; compared with the corresponding dose of the China-approved recombinant collagen, P > 0.05.
